# The Challenges and Relevance of Exploring the Genetics of North Africa's “Barbary Lion” and the Conservation of Putative Descendants in Captivity

**DOI:** 10.1155/2016/6901892

**Published:** 2016-08-30

**Authors:** Simon A. Black

**Affiliations:** Durrell Institute of Conservation and Ecology, School of Anthropology and Conservation, University of Kent, Canterbury, Kent CT2 7NZ, UK

## Abstract

The lions of North Africa were unique in ecological terms as well as from a human cultural perspective and were the definitive lions of Roman and Medieval Europe. Labelled “Barbary” lions, they were once numerous in North Africa but were exterminated by the mid-20th century. Despite subsequent degeneration of the Atlas Mountain ecosystem through human pressures, the feasibility of lion reintroduction has been debated since the 1970s. Research on the long-established captive lion collection traditionally kept by the sultans and kings of Morocco has enabled selective breeding coordinated across Moroccan and European zoos involving a significant number of animals. Molecular genetic research has recently provided insights into lion phylogeny which, despite previous suggestions that all lions share recent common ancestry, now indicates clear distinctions between lions in North, West, and Central Africa, the Middle East, and India versus those in Southern and Eastern Africa. A review of the evolutionary relevance of North African lions highlights the important challenges and opportunities in understanding relationships between Moroccan lions, extinct North African lions, and extant lion populations in India and West and Central Africa and the potential role for lions in ecosystem recovery in those regions.

## 1. Introduction

Lions (*Panthera leo*) formerly ranged throughout Africa, the Middle East, and southwestern Asia [1–4]. In recent prehistory up to 2500 BC, lions persisted in Europe as far north as Bulgaria, Hungary, Ukraine, Italy, and Spain with some still sighted in Greece into early historic times before their eventual extinction [5, 6]. Populations found at extremes of the range were traditionally considered distinctive geographic groups and given colloquial names including the “Cape lion” in South Africa, the “Barbary lion” in North Africa (Morocco, Algeria, Tunisia, and Libya), and the “Asiatic lion” in the Middle East through India [4]. The Asiatic lion is currently the only population listed as a subspecies (*P. l. persica*) by the IUCN and CITES [7] although the species taxonomy for lions is under review by the Cat Classification Task Force of the IUCN SSC Cat Specialist Group [3].

**Figure 1:**
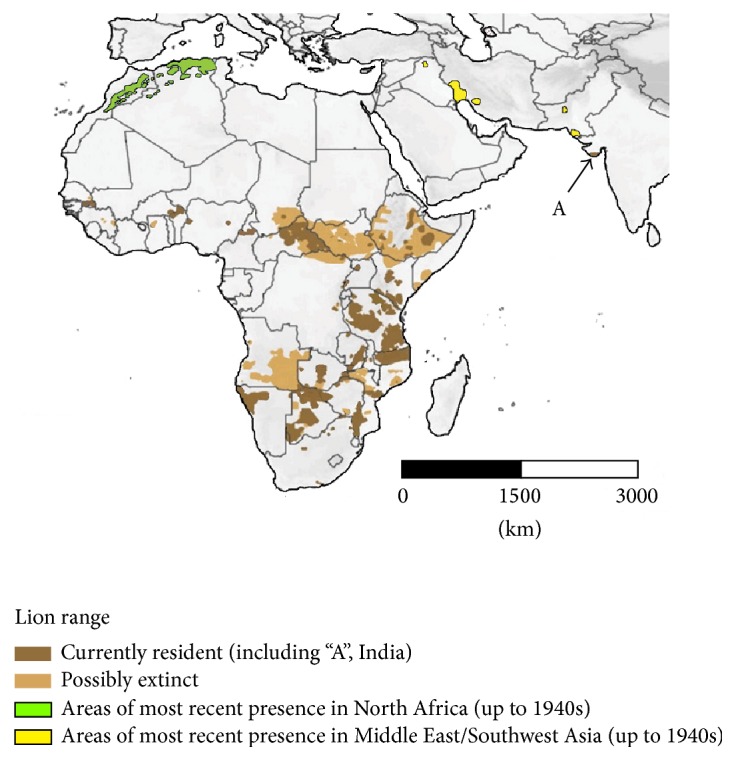
Current and most recent historical distribution of lions (*Panthera leo*) in sub-Saharan Africa and India adapted from analysis of known lion habitats [11], plus locations of now-extinct 20th century remnant populations in North Africa, the Middle East, and Pakistan [2, 5].

The range and population size of lions have significantly declined over the past 150 years. South African Cape populations were extinct by the mid-19th century and North African lions had disappeared by the mid-20th century [2, 8, 9]. The Asiatic lion disappeared from Syria, Turkey, and Iran in the 19th century, held on longer in Iraq until the 20th century [5, 6], and, despite approaching extinction, still survives as a population of several hundred animals in the Gir Forest and adjacent pockets of habitat down to the shores of the Arabian Sea in the state of Gujarat, India [7, 10]. Today, West and Central African lion populations survive in small isolated groups and have declined significantly in recent decades [3]. The last strongholds for lions are in Eastern and South-Eastern Africa [3, 11] (Figure 1).

Traditionally, the subspecies status of populations of lions was determined by hunters, naturalists, and scientists, largely on the basis of morphology and geography [4], and consequently the historical taxonomy of* Panthera leo* has previously been quite complex, only now becoming clearer through recent advances in molecular research [12–14].

The colloquially named “Barbary” lion of North Africa was familiar to historical writers in Europe due to the proximity of wild populations. The transport of wild lions to gladiatorial events of the Roman period and later menageries of medieval Europe imprinted the Barbary lion in popular culture, art, and literature [15]. The morphology of those animals, the shaggy mane and forequarters, became the model for lion images in mosaics, heraldry, pottery, paintings, and sculpture. North African lions are intriguing to scientists, natural historians, and conservationists being perceived through morphology and behavioural ecology as the most distinctive of all lion populations [15, 16], living in temperate, forested montane habitats of North Africa's Maghreb region [2].

The North African Maghreb is the region isolated from the rest of nonarid Africa by the Sahara and forms the southern extent of the Mediterranean biodiversity hotspot [17]. This is a region of high species endemism as well as an unusual mix of African and Eurasian species; however, lions have not existed in the wild in North Africa since the late 1950s [2, 8]. Whilst the habitat in the Maghreb is now thought to be largely unable to support the needs of lions, leopards still appear to survive in the region [18]. The interest in a potential reintroduction of lions to North Africa carries a broad conservation [16, 19, 20] and scientific [8, 17] and cultural significance [19, 21], and the topic regularly surfaces both within conservation circles and the wider media [15, 22–25].

The North African “Barbary” lion's recent extinction history and the impact of human encroachment into its former range have been well documented in the literature [2]. The loss of lions from North Africa repeats a similar pattern of anthropogenic decline observed in Europe in ancient historical times (including across the Caucasus and areas around the Caspian Sea) and later through the countries of the eastern Mediterranean and across the Middle East since medieval times [2, 6]. The North African lion decline also resonates with the challenges faced today by dwindling lion populations in West and Central Africa [11, 26, 27], where a small, dispersed population of around one thousand animals is perilously close to extinction, including several extremely vulnerable isolated subpopulations of perhaps ten or twenty animals approaching imminent demise [26, 27]. An examination of the significance of North Africa's “Barbary” lion, its relevance to global lion conservation, and potential place in contemporary conservation of North Africa and the wider continent is overdue.

## 2. The Biogeographical and Ecological Significance of the Barbary Lion

Scientific understanding of how the various lion populations are related is now becoming clearer, based upon the findings from recent molecular genetic studies [12, 14, 28]. North African (“Barbary”) lions were previously connected to other populations through the Mediterranean basin and the Nile delta eastwards and transient corridors south occurring during changes in Saharan climate [25] but ultimately became separated by the Sahara desert to the south and the later rise of the Egyptian empire to the east 4000 years ago [9]. The historical North African lion is, however, an important ecological link between the last surviving lions in India and the dwindling West and Central African populations [12].

**Figure 2:**
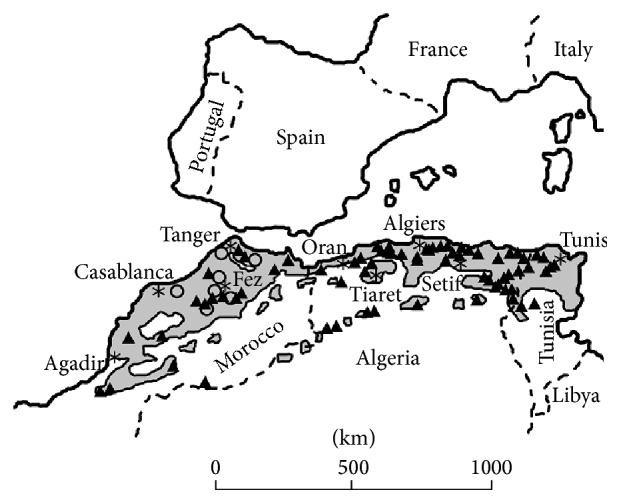
Documented lion sightings since the middle ages across the Maghreb biome of the southern Mediterranean (light grey shading) north of the Sahara in North Africa (AD1500–1960). Open circles depict locations of general historical observations documented before 1800, adapted from [2]. Details can be sourced from [2, 8, 15]. Asterisks denote the locations of the various named major human population centres.

Aside from earlier recorded histories of the Roman and medieval periods, sightings of lions in North Africa were regularly documented by travellers from the 16th century [2] in the lowland northern coastal plains and forests and the Rif Mountains of Morocco as well as the coastal forests of Algeria and Tunisia (Figure 2). As 19th century travellers later ventured into remoter areas such as the Tell Atlas of northern Algeria [29], the higher altitudes of the Middle and High Atlas mountains of Morocco [30], and further south into the Saharan Atlas range [4] and into remoter Saharan fringes [31], lions were encountered and found to be familiar to local people [2]. Land encroachment by human communities from the 1600s across northern Libya, Tunisia, Algeria, and Morocco led to the final extirpation of lions from North Africa by the mid-20th century [2, 8].

Lions finally disappeared from the Maghreb due to a combination of human pressures on the landscape and direct extirpation [2], a loss which occurred, therefore, just a few decades before the emergence of the modern postcolonial period of wildlife conservation. The possibility of restoring the animal back to the region has nevertheless been debated at least since the 1970s [16, 22, 25]. To justify and support lion conservation management by way of translocations, reintroduction, or captive management, it is important to define distinctive populations of animals, also referred to as evolutionary significant units or ESUs [1]. Recent molecular advances have enabled traditional taxonomic classification of lion subspecies to be simplified significantly. The latest studies suggest to the IUCN that* Panthera leo* should be split into two groups; the first is wild populations found (whether in part, formerly, or currently) in North Africa/Asia, West Africa, and Central Africa, which should be considered* Panthera leo leo*, whilst the second group in Southern Africa, East Africa, and North East Africa should be considered* Panthera leo melanochaita* [32]. At the very least, each population within these groups should be managed as an evolutionary significant unit (ESU) [11, 12, 33].

In general terms, historical populations of North Africa's “Barbary” lion would therefore be considered within the modern taxonomy for* Panthera leo leo* and within this context considered as an evolutionary significant unit (ESU) based on geographical location. In the absence of a wild population in North Africa today, contemporary conservationists argue that lions should be restored as an apex predator to balance the ecosystems of the region. Potential candidates for reintroduction would include either their nearest relatives from India [12] or possible putative “Barbary” lions in captivity should they be proven to be authentic.

## 3. Possible Extant Captive Representatives of the North African “Barbary” Lion

Despite the disappearance of animals in the wild, debate surrounding the possibility of extant representatives of North African lions in captive collections has continued for decades without resolution. In Morocco, a collection of lions, traditionally trapped and presented to the Sultan by the local Berber tribes who inhabited the surrounding region, have been held at the Royal Palaces for centuries [15]. It is not definitely known whether today's descendants of these animals (hereafter termed “Moroccan lions”) represent the wild North African lion or only partly descended from wild North African stock or are indeed completely unrelated to wild North African lions.

Previous attempts to address the issue of identification were led in the 1970s by Helmut Hemmer and Paul Leyhausen [22]. In the absence of pedigree information, Hemmer and Leyhausen instigated a breeding programme for Moroccan lions to select against foreign allele introgression by determining external characteristics and by checking skull features in respective breeding lineages [16]. Whilst historical accounts of North African “Barbary” lions often paid attention to the luxuriant mane and other morphological features, there is no strong evidence that these particular traits were definitive [4, 15]. Cases of morphological identification of captive lions are confounded by the effects of nutrition, testosterone levels, abrasion or wear, and climatic effects on mane growth. It has been suggested that these variables could contribute up to half of the observed variation in manes [34].

In the late 1980s when genetic research appeared to suggest that all lions (including Asiatic lions) shared a common ancestor, many zoos stopped their involvement in captive conservation efforts with Moroccan lions [25] and several zoos which previously bred Moroccan lions abandoned their programmes, as a result of difficulties in obtaining new breeding stock (A Harland pers. Comm.). Nevertheless, aside from the animals that descended from those known to Hemmer and Leyhausen [22], other captive lions suspected of being of direct North African ancestry have since periodically surfaced in zoo collections and circuses despite the lack of evidence to confirm whether these captive animals are authentic representatives. Fairly recent claims have since been made for the “Barbary” origins of captive lions in Addis Ababa zoo [35], using morphological comparisons (mane size and appearance) noting the possibility that their forebears were a gift from the King of Morocco to the Emperor of Ethiopia. Interestingly, recent genetic work suggests that these captive Ethiopian lions are actually a distinct subpopulation in their own right [36].

The most plausible prospect is that animals derived from the King's collection in Morocco are the most likely wild descendants from North Africa [37]. Although zoo-based efforts to breed Moroccan lions were temporarily halted in the 1980s and 1990s, the work was revived and improved in 2009 with the development of a scientifically based studbook breeding programme in European zoos [37]. This work stimulated interest amongst zoos to transfer and breed the animals, establish new collections, and enable the captive population to survive [2]. The captive population has since demonstrated improved breeding success [38] and several zoos have new breeding groups.

However, there is a possibility that some individuals in the original Moroccan collection bred with lions of non-North-African descent before the 1960s (A. Harland pers. comm.; W. Frey pers. Comm.). Whilst this raises the chance of a watered-down genotype across the current captive population, this hypothesis is currently untested and the facts are therefore unknown. In the absence of definitive diagnostic molecular genetic techniques, including investigation of paternal bloodlines, neither the distinctiveness of Moroccan lions (or indeed any other candidate animals) nor their authenticity as true North African lions has been established [39]. Nevertheless, if the non-North African animals were close relatives of lions from India or West or Central Africa, their offspring would remain authentic representatives of* Panthera leo leo* within the IUCN's proposed classification [3].

If Moroccan lions were genetically designated within* Panthera leo leo* (West Africa, Central Africa, North Africa, and India), then as a captive group they become significant. The current known population for the* P. l. leo *group, accounting for known Asiatic lions in captivity (N, circa 100 [3]), plus 400 in the wild in India itself [7], with perhaps 250 individuals in West Africa [3, 40] and around 1000 in Central Africa [11, 41], forms a total of perhaps 1750 individuals worldwide under the proposed designation of* Panthera leo leo.* The additional captive Moroccan lions (N = circa 100 individuals) would represent a significant proportion (>5%) of the extant genome for the designated subspecies. This captive Moroccan lion group is also unusual since the original menagerie curators and subsequent zoos have kept the animals separate from generic zoo lions (i.e., other lions largely of East African descent) for nearly 50 years [37].

## 4. Lion Phylogeny and the Place of North African Lions

Currently, the understanding of genetics in lions lags somewhat behind equivalent knowledge for leopards, jaguars, and tigers [1]. Historically, the study of lions was largely focused on qualitative assessments of morphology. It is possible that genetic divergence between lion populations was accelerated due to isolation caused by geographical changes and human habitation [1], but evidence suggests that traditionally observed variation in morphology is greater in larger bodied mammals [42], and phenotypes (both morphology and behaviour) may be affected by genetic drift, such as the increasing presence over the last 150 years of certain cranial characteristics in Asiatic lions.

**Figure 3:**
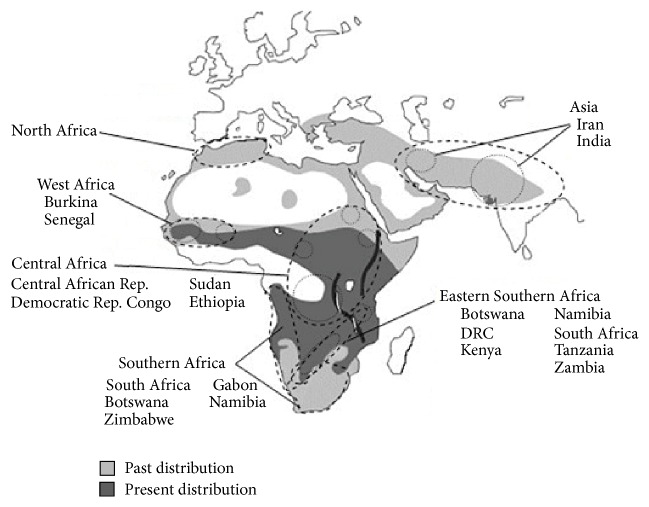
Approximate geographic distribution of unique mtDNA haplotypes in lions from control region (HVR) and cytochrome b (adapted from [12, 45]). Source countries for sampled lions of known origin are listed. The Great Rift Valley is shown as thick dark lines.

The past decade has seen increasing clarity on the relationships between lion populations through the application of molecular techniques. Initial progress was made by Barnett et al. [43–45] using the mitochondrial cytochrome b region (which codes for an enzyme involved in the respiratory chain complex) and a short 130 bp strand of mtDNA in the hypervariable noncoding control region. The latter is useful in retaining genetic variation since it is a nonfunctioning DNA sequence unaffected by selective pressures. The identified haplotypes generated a phylogeographic distribution of lion populations, based on 32 origin-known lions (both contemporary and ancient museum specimens). Modern lions showed 13 variable sites across the control region enabling reconstruction into phylogeographic groups including West Africa, eastern Sahel (steppe/savannah areas south of the Sahara), Eastern-Southern Africa, Southwestern Africa, and North Africa-Asia (Figure 3).

Microsatellites (noncoding, repeating variable patterns of DNA base pairs) have also been used alongside mtDNA analysis in a number of studies to differentiate lion populations and infer evolutionary history of the species [14, 46, 47].

Improvements in molecular technologies have, however, encountered a number of practical limitations including a lack of reference samples or the necessity to sample captive animals of dubious provenance [47]. First, the reliance on maternally transferred haplotypes in samples of suspected nontarget origin continues to confound a complete genomic understanding. Secondly, the earlier microsatellite studies of samples from across Africa and India [44] included animals of putative North African origin but lacked samples from West Africa. Even Antunes et al.'s [46] promising recent study of genetic information left by lion feline immunodeficiency virus (FIV_Pleo_) within host lion populations could only consider sub-Saharan African lions, since Asiatic lions were found not to have the virus and ancient samples from North Africa were not accessed.

The most recent studies have been able to move towards a clearer consensus, particularly that extant populations of lions from West and Central Africa (plus India) should be considered distinct from lions in Southern and East Africa [13, 14]. Dubach et al.'s [28] extensive study generated a phylogeny with lions grouped into two well-supported clusters: North, West, and Central Africa plus India (6 haplotypes) and Eastern and Southern Africa (14 haplotypes). The former group was itself subdivided into three clusters: the Indian Gir Forest, North and Central Africa, and West Africa. Lions from Eastern and Southern Africa were collectively more weakly clustered together, for example, lions from Botswana appearing in both Eastern and Southwestern clusters. This work was able to identify a stronger basis for considering the genetic relatedness of lion populations when considering and enacting translocations as part of active population management. Furthermore, the study highlighted the extensive natural movement of lions between Eastern and Southwestern clades and its implications on conservation management in those regions. From this, Dubach et al. (2013) [28] proposed that there were two principal clades of lions which should be recognized as* P. l. leo* and* P. l. melanochaita*.

Bertola et al. [13, 14] conducted further analysis of over 100 samples across the species' range, to generate a phylogeny that also challenges the traditional taxonomic split of African lions and an Asian subspecies. They further argue, in an analysis which builds on historical studies by earlier authors [12, 45], that the traditional taxonomy does not satisfactorily reflect the overall genetic diversity that can be observed across modern lion populations [14]. Bertola et al. [14] additionally identified emerging consistencies in phylogeographic patterns described by both autosomal markers and mtDNA studies suggesting genetic lineages which should be prioritised to conserve genetic diversity [13, 47]. Their analysis of 20 microsatellites and 1,454 bp of mitochondrial DNA in 16 lion populations across the entire geographic range of the species identified four major clusters from both types of markers: West/Central Africa, Eastern Africa, Southern Africa, and Asia/North Africa [14, 47].

The pattern of clusters identified in these extensive population genetics studies suggests a possible ice age extinction of lions from West and Central Africa, followed by recolonisation of the region by lions from refugia in the Middle East [14, 21]. In effect, this creates a taxonomic split into two subspecies: a north group of* P. l. leo* (India, West Africa, and Central Africa) and a south group of* P. l. melanochaita* (Southern Africa, East Africa, and North East Africa) [12, 14, 28, 32, 47]. This revised subspecies taxonomy is currently under consideration for formal adoption by the IUCN [3].

## 5. Genetic Investigation of the Moroccan Royal Lion Collection

Previous research including genetic analysis infers that lions from the Rabat Zoo supposedly descending from Barbary lions have a complicated history including a suggestion that they originated in West or Central Africa instead [12, 14, 28, 43]. Barnett et al. [45] suspected that a Central African haplotype in many captive lions of supposed Moroccan origin was due to introgression. An alternative, untested, and least likely hypothesis is that the remaining animals derived from the Moroccan Royal collection were never of North African origin (somewhat improbable due to the particular efforts made to keep them as a distinct group over centuries and certainly over many recent decades). A third, untested but relevant possibility is that Moroccan lions carry haplotypes consistent with Central Africa simply because they are historically part of the same broad population (i.e., North Africa, West Africa, Central Africa, and India). This latter situation is currently difficult to test since the number of museum reference samples is so small and therefore less likely to be representative of all haplotypes present across the original North African population.

So whilst mitochondrial DNA studies on a small number of animals from the Moroccan Royal Lion collection have demonstrated that these individuals cluster with lions from Central Africa [12, 13], an additional word of caution should be expressed. The studies are to date based on tiny samples of particular individuals and not a systematic sampling of all Moroccan lions across all bloodlines in the Moroccan lion studbook. The studbook includes 12 founder bloodlines across the whole captive population [37], yet only two or three of these bloodlines have been analysed in DNA studies to date [12, 13, 21, 43, 46]. Also, since mitochondrial DNA expresses maternal lineage only, any retained paternal genotype that could include North African heritage remains undetected.

The number of Moroccan lions (all held in captivity) is under 100 animals, well below the 1000 or so individuals which is regarded as the lower limit for an effective, sustainable population. Moroccan lions as a group are therefore vulnerable to the normal threats associated with small populations [48], amplified by the restrictions of the dispersed captive environment. In terms of population management, expansion of the captive breeding project reduces these risks as well as maintaining appropriate gender balances for future breeding. An additional complication occurs where some groups are dominated by particular breeding pairs resulting in lack of normal pair selection that would be experienced in the wild [37], causing a potential skew in the genetic structure of the population.

The Moroccan lion group has likely undergone a genetic bottleneck, due to the establishment (or sometimes historic shrinkage) of the population to a small number of individuals from which the subsequent existing population is founded, largely imposed by the limitations of captive breeding. Within a bottlenecked population, inbreeding may have occurred which could skew genetic characteristics (“genetic drift”) due to limitations in parental heritage [49]. This could explain differences in genetic traits if a “one-off” haplotype is retained in the population due to the imposed breeding bottleneck whereas in a larger population the genotype might otherwise dissipate and possibly disappear. Another possibility is that introgression with introduced lions from outside Morocco has caused expression of a haplotype for another geographical region which is, as yet, not identified within literature. For example, Barnett et al. [43] concede that to date little data exists for other populations of lions from the Sahel (i.e., the immediate Sub-Saharan region).

Burger and Hemmer [50] report an analysis of cytochrome b extraction for a cub from Neuwied zoo, Germany, born of parents taken from the Moroccan collection. Their genetic results suggest this individual is more similar to Asiatic lions (*P. l. persica*) than Sub-Saharan lions. However, Yamaguchi [39] argues that, in the absence of a comparison of this sequence with ancient DNA from known Barbary lion specimens, it is difficult to conclude that the Neuwied zoo cub is true “Barbary” (i.e., of direct wild North African descent). Also, the comparison sequences used by Burger and Hemmer [50] did not include lions representing regions such as the Sahel, so the difference in the cub could be due to non-North African genotype.

Variation in cytochrome b (an mtDNA region of approximately 1300 base pairs) is very low across lion populations as would be expected, since the region codes for a protein necessary for electron transport within cells so variation over time (mutation rate) is very low. Cytochrome b is commonly used in phylogenetic reconstruction and is useful in initial matching of samples with lion mtDNA documented in GENBANK [51] and has enabled various lab protocols to be established and the outputs to be verified. To date, control region sequence has been essential to explore regional variations in origin-known lions, due to it being a noncoding mtDNA region and therefore able to accumulate greater mutational variation without being purged from the genome due to deleterious effects. Although haplotypes in a 130-base pair strand of control region have been used as identifiers of differentiation in lion populations of known origin [12, 43, 44], emerging genomic analyses are likely to provide a better understanding of taxonomic differences in the future.

## 6. Discussion

The North African “Barbary” lion remains an enigma, recently extirpated, less accessible even to established ancient molecular genetic techniques (due to the small number of available samples), and tantalisingly out of reach to modern conservation interventions. Clearly, as apex predator, the species had an important role in North African ecosystems and its cultural importance within North African countries and their near neighbours around the Mediterranean suggests that the lion is a potential flagship for conservation of the Maghreb region. To date, the insufficient evidence to count or discount the relevance of putative descendants, particularly the captive Moroccan Royal Lions, does not change this expectation.

The evolutionary importance of finding representatives who could form the basis of a recovered North African population is significant, since the only other representatives of the “northern” lion clade (which the IUCN [3] suggests as* Panthera leo leo*) are either the few hundred Asiatic lions living wild in Gujarat or those captive in Asiatic lion studbook programmes or the few hundred wild animals dispersed at low densities across West and Central Africa. These highly threatened populations, particularly the micropopulations in West and Central Africa, form a group that is distinct from the bulk of global lion populations, found in Eastern and Southern Africa [12, 14, 32, 43, 45, 47], so active conservation planning and systematic intervention for the former group are in any event paramount.

Next-generation sequencing methodologies offer opportunities to examine single nucleotide polymorphisms (SNPs) between members of a species. SNV/SNP genotyping techniques may hold an important role in determining the lion genome to an extent where regional populations can be identified as has been demonstrated in other fields [52]. Important early SNP work to date with lions by Bertola [47] has so far confirmed the proposed north/south division in lion classification which has been proposed to the IUCN [3]. SNP methods have also been applied in other felid populations that comprise potentially introgressed individuals [53] providing opportunity to “weed out” introgressed individuals from the Moroccan lion population. SNP techniques have already been used as part of local population management and breeding control in captive lion collections against degenerative diseases [54]. Although viral DNA analyses of the type used in FIV analyses [46] offer potential to overcome some of these shortfalls, the ancient reference samples held in museum collections might not offer suitable viral material within lion samples for application of those techniques. Since no lions exist in the wild in North Africa today, the essential limiting issue with all of these advanced genetic techniques is the reliance on access to recovered DNA from verified origin-known ancient museum specimens, which are themselves extremely rare.

As Yamaguchi [39] points out, currently, there are no possibilities of identifying the true status of living putative representatives of the North African lion without either (i) comparison directly to lions of known North African origins (through ancient DNA from museum samples) or (ii) comparison with samples from all extant lion populations. Although mitochondrial DNA analyses continue to provide insights into the evolutionary history of lions and regional variances [43, 45], these diagnostic tools are limited to the maternal line of descent, the differentiating haplotypes being sourced in mitochondrial DNA. Examination of paternal measures of biogeographical origin in lions (through genomic DNA) will be important for future research investigation. Although better diagnostic examination of lion clades can be made by further exploring mitochondrial DNA building on haplotypes identified by Barnett et al. [12, 43, 45], future use of genomic DNA is more likely to enable significant progress in differentiating lion genotypes [47].

In terms of the captive Moroccan lion population, to date, the findings from a small number of mtDNA isolates suggest that the genetic characteristics of Moroccan lions contrast with other lions of known origin [12, 50]. Further exploration should be conducted to validate these assertions and to compare results with a wider sample of individual lions. Yamaguchi [39] notes the importance of* ex situ* conservation of the lion to support retention of its genetic diversity. For example, the mtDNA haplotype within cytochrome b of the Neuwied Moroccan Royal lion is not found in any wild lion population so far tested (only in one origin-unknown zoo lion). It is possible therefore that genetic characteristics lost from wild populations may continue to survive in captive animals. This increases the potential importance of restoring the zoo population of Moroccan lions and better understanding of their genetics relative to wild lion populations and the conservation management implications of that knowledge.

In any event, lion populations are in dangerous decline across their range, with the IUCN suggesting a 43% population decline between 1993 and 2014 [33] noticeable even in the past 10–15 years, most particularly in West Africa [41]. It is a remarkable, albeit somewhat uncomfortable, fact that the 90-or-so Moroccan Royal Lions recorded in the EAZA studbook represent a population in the same order of magnitude as the global captive Asiatic lion studbook population, perhaps only one-half the size of the wild population in West Africa and one-quarter the size of the wild population in India [37]. The preservation of the IUCN designated* Panthera leo leo* ESUs [3] and the entire lion genome globally (both* in situ* and* ex situ*) is vitally important in the species' future conservation [55]. On a practical level, the small size of the target zoo population of Moroccan lions means that, to preserve genetic variation, a key requirement will be to equalise founder representation and maximise effective population size [56] and genetic studies will help to determine these decisions.

However, the potential restoration of an authentic North African “Barbary” bloodline as an ESU, through some form of backbreeding of Moroccan lions, might itself also be possible and is not entirely without precedent. The approach shows similarities to the process already used for Przewalski's horse [15]. Recent developments in molecular methodologies mean that genetic approaches could support selective breeding if sufficient genetic characteristics can be defined for the true North African (“Barbary”) genotype [12]. Nevertheless, if the breeding of Moroccan lions in captivity is deemed worthwhile, by inference and precedent in conservation practice, that breeding programme should be purposed towards the ultimate reintroduction of the animals into the wild [57]. This could ultimately influence the preservation of lions across their precarious northern ranges in India, West Africa, Central Africa, and, potentially, North Africa itself.

## 7. Conclusions

If potential representatives of North Africa's “Barbary” lion still persist in zoo collections, then efforts to conserve its genotype are justified. Provisional research should focus initially on which origin-known haplotypes [12, 45] can be identified within Moroccan lions, particularly the various bloodlines in that small population [37]. It may also be prudent to investigate the collection of lions at Addis Ababa and other putative “Barbary” lions [36] as these may prove to be closely related to the last lions of West and Central Africa and India. More importantly, future genomic investigations should explore the relative relatedness of captive Moroccan lions versus wild lion groups.

In terms of* in situ* conservation of lions in North Africa, it is also possible to consider [12] whether India's “Asiatic” lions should be treated as conspecific with North Africa's “Barbary” lions and therefore propose the option that Asiatic lions could be used in a future North African reintroduction. Precaution would suggest that the status of putative “Barbary” lions, such as Moroccan lions, should be definitively established (or rejected) from comprehensive genetic understanding of the genome before turning to this alternative source of animals. Nevertheless, taking a wider perspective on global lion conservation, the possibility of translocating Indian animals to a suitable location in North Africa would offer the advantage of a second wild population of the endangered Asiatic lion as representatives of the North African-Asian clade. If Moroccan lions are shown to be relatives of Indian, West African, and Central African lions, then a new perspective can be taken on their potential use as a genetic pool to support recovery in those regions with the most threatened populations of* Panthera leo*.
